# The Effectiveness of Ultraviolet-C (UV-C) Irradiation on the Viability of Airborne *Pseudomonas aeruginosa*

**DOI:** 10.3390/ijerph192013706

**Published:** 2022-10-21

**Authors:** Thi Tham Nguyen, Congrong He, Robyn Carter, Emma L. Ballard, Kim Smith, Robert Groth, Esa Jaatinen, Timothy J. Kidd, Thuy-Khanh Nguyen, Rebecca E. Stockwell, George Tay, Graham R. Johnson, Scott C. Bell, Luke D. Knibbs

**Affiliations:** 1 School of Public Health, The University of Queensland, Brisbane, QLD 4006, Australia; 2 International Laboratory for Air Quality & Health, School of Earth and Atmospheric Sciences, Faculty of Science, Queensland University of Technology (QUT), Brisbane, QLD 4000, Australia; 3 Centre for Children’s Health Research, Brisbane, QLD 4101, Australia; 4 QIMR Berghofer Institute of Medical Research, Brisbane, QLD 4006, Australia; 5 School of Chemistry and Physics, Queensland University of Technology (QUT), Brisbane, QLD 4000, Australia; 6 School of Chemistry and Molecular Biosciences, The University of Queensland, Brisbane, QLD 4032, Australia; 7 Pathology Queensland, Royal Brisbane and Women’s Hospital, Brisbane, QLD 4006, Australia; 8 The Prince Charles Hospital, Brisbane, QLD 4032, Australia; 9 Faculty of Medicine, The University of Queensland, Brisbane, QLD 4006, Australia; 10 Translational Research Institute, Brisbane, QLD 4102, Australia; 11 Public Health Unit, Sydney Local Health District, Camperdown, NSW 2050, Australia; 12 Faculty of Medicine and Health, School of Public Health, The University of Sydney, Sydney, NSW 2006, Australia

**Keywords:** airborne bacteria, inactivation rate, air disinfection, *Pseudomonas aeruginosa*, ultraviolet light, UV-C radiation

## Abstract

*Pseudomonas aeruginosa* (*Pa*) is the predominant bacterial pathogen in people with cystic fibrosis (CF) and can be transmitted by airborne droplet nuclei. Little is known about the ability of ultraviolet band C (UV-C) irradiation to inactivate *Pa* at doses and conditions relevant to implementation in indoor clinical settings. We assessed the effectiveness of UV-C (265 nm) at up to seven doses on the decay of nebulized *Pa* aerosols (clonal *Pa* strain) under a range of experimental conditions. Experiments were done in a 400 L rotating sampling drum. A six-stage Andersen cascade impactor was used to collect aerosols inside the drum and the particle size distribution was characterized by an optical particle counter. UV-C effectiveness was characterized relative to control tests (no UV-C) of the natural decay of *Pa*. We performed 112 tests in total across all experimental conditions. The addition of UV-C significantly increased the inactivation of *Pa* compared with natural decay alone at all but one of the UV-C doses assessed. UV-C doses from 246–1968 µW s/cm^2^ had an estimated effectiveness of approximately 50–90% for airborne *Pa*. The effectiveness of doses ≥984 µW s/cm^2^ were not significantly different from each other (*p*-values: 0.365 to ~1), consistent with a flattening of effectiveness at higher doses. Modelling showed that delivering the highest dose associated with significant improvement in effectiveness (984 µW s/cm^2^) to the upper air of three clinical rooms would lead to lower room doses from 37–49% of the 8 h occupational limit. Our results suggest that UV-C can expedite the inactivation of nebulized airborne *Pa* under controlled conditions, at levels that can be delivered safely in occupied settings. These findings need corroboration, but UV-C may have potential applications in locations where people with CF congregate, coupled with other indoor and administrative infection control measures.

## 1. Introduction

Cystic fibrosis (CF) is the most common life-limiting autosomal recessive genetic disorder in Caucasians and is caused by variants in the cystic fibrosis transmembrane conductance regulator (CFTR) gene. Chronic lung infection and associated inflammatory response results in damage to airways and lung tissue and is the leading cause of morbidity and mortality in CF. The prevalence of *Pseudomonas aeruginosa* (*Pa*) increases with age and ~70% of adults with CF have chronic *Pa* infection [1,2]. Other common bacterial pathogens in people with CF include *Staphylococcus aureus*, Gram-negative non-fermentative bacteria (such as *Burkholderia cepacia* complex, *Stenotrophomonas maltophilia*, *Achromobacter xylosoxidans)* and non-tuberculous mycobacteria (most commonly *Mycobacterium avium complex* and *Mycobacterium abscessus*) [3,4]. Life expectancy with CF has increased progressively over recent decades (median survival in most countries now approaches or exceeds 50 years [5,6,7,8]). Consequently, specialized CF clinical care centers are now standard for people with CF.

While the exact mechanisms of cross-infection between people with CF remain unknown, the potential for person-to-person transmission of common CF pathogens amongst people attending the same center has become evident and policies and practices to limit *Pa* transmission in health care settings (and other indoor settings) where people with CF congregate have been developed. Such approaches include single occupancy inpatient accommodation, segregation of patients attending outpatient clinics based on airway microbiology and the use of face masks as a routine when people with CF attend the hospital of clinical care [9,10]. Although contact and droplet infection control measures were implemented [9,11], it is likely that cross-infection continues [12,13,14]. While contact and large droplet transmission (i.e., transmission by respiratory droplets ≥ ~20 μm in diameter and typically within <1 m) modalities are important, several recent studies have shown that airborne transmission (i.e., by viable particles ≤ ~5–10 μm in diameter persisting in the air for long durations) is one of the potential routes for infection in CF, which was previously assumed not to occur [15,16].

Recent evidence from our group and others has suggested transmission by droplet nuclei (i.e., aerosol transmission) can occur for *Pa* and other common CF pathogens [17,18,19,20]. For example, our previous studies have demonstrated that patients with CF produce viable aerosols of *Pa* [17] and when people with CF cough, viable *Pa* can travel 4 m and survive for up to 45 min in room air [18]. Those findings were not consistent with CF infection control practices that separated patients by 1 m to 2 m to prevent cross-infection [9,21]. Moreover, those findings have raised questions about the type and efficacy of interventions to limit transmission of airborne *Pa* in healthcare settings. In follow-up studies, we found that such methods, like mechanical ventilation [22], and surgical masks and N95 respirators, are useful, but cannot completely capture airborne *Pa* (e.g., surgical masks can reduce airborne *Pa* to below detectable levels in 55–89% of adult wearers who produced culture-positive samples when not wearing the mask [23,24]), nor are they feasible in all clinical settings (e.g., performing airway clearance/physiotherapy and lung function testing) and patient groups (e.g., the effectiveness of face masks among children is unknown) [18,24,25].

Germicidal irradiation with ultraviolet (UV) light within the band from 200–280 nm (UV-C) has long been known to reduce microbes in indoor air because it damages their DNA and prevents replication [26]. UV-C in 250–270 nm range is the most potent wavelength for inactivating microbes since it is more strongly absorbed by their nucleic acids [27,28]. Rapid inactivation of airborne *B. coli* by UV-C was initially observed by Wells and Fair in 1935 [29]. Subsequently, laboratory studies performed under controlled conditions, have determined the effectiveness of UV-C for various aerosolized microorganisms [30,31,32,33,34,35,36,37,38,39,40]. Several factors are reported to affect indoor UV-C effectiveness, including UV-C intensity (units: μW/cm^2^), UV-C dose (intensity × time [s], units: μW s/cm^2^), the susceptibility of a given microorganisms, photoreactivation, relative humidity (RH), temperature, room air exchange rates, and air mixing [26,41,42,43].

We recently undertook a systematic literature review [44] and found 17 studies of UV-C effectiveness for airborne respiratory pathogen bacteria. In that review and subsequent literature searches, we identified only three previous empirical studies of the UV-C susceptibility of airborne *Pa*, the first of which was published in 1940, and all of which used small experimental chambers (of the order of ~10 to ~30 cm in any direction) and artificially generated aerosols [34,45,46]. Another study used a theoretical model to investigate the air disinfection efficacy of duct-mounted UV-C lamps in a hospital where mechanical ventilation is present [47]. However, that study was based on UV-C inactivation values for *Pa*, previously published by Collins [48], and which were for *Pa* on surfaces (agar plates), rather than in suspended in air. The three empirical studies support the hypothesis that airborne *Pa* are susceptible to UV-C (i.e., that *Pa* inactivation with UV-C on is greater than that observed without UV-C). However, it is unclear these observations are applicable to *Pa* strains in the CF community, and whether doses reported are achievable in occupied healthcare settings given safety requirements for patients, visitors and staff.

We aimed to quantify the effectiveness of UV-C on aerosolized *Pa* at doses appropriate for potential clinical settings attended by people with CF, under a range of experimental conditions, and estimate if the doses can be implemented safely in an occupied indoor clinical setting.

## 2. Materials and Methods

### 2.1. TARDIS-Rotator and UV-C System

**Figure 1:**
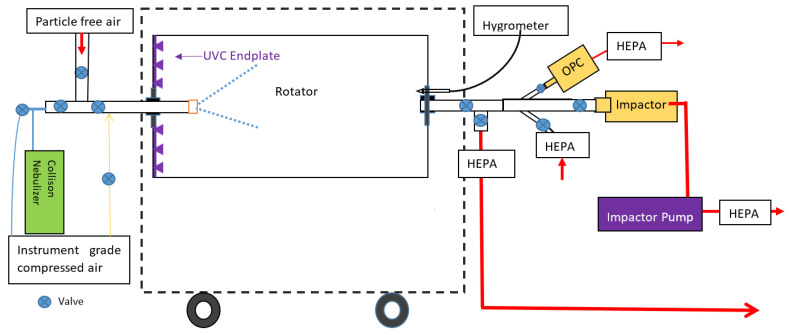
Schematic diagram of equipment and *Pa* nebulization and UV-C treatment. Note, the LEDs were mounted on the left end plate of the rotator. The rotator is 1.2 m long with diameter: 0.65 m (volume: 400 L).

We used a custom-built rotating drum sampler (Tandem Aged Respiratory Droplet Investigation System, TARDIS), which allows storage and aging of nebulized airborne *Pa* [18,49], as previously described. Briefly, the TARDIS is a fully sealed, horizontally oriented rotating cylindrical stainless steel drum (length: 1.2 m, diameter: 0.65 m, volume: 400 L), fitted with inlet and outlet ports that has been described in detail previously; Figure 1 is a schematic diagram [49]. The TARDIS rotator is flushed with high-efficiency particulate air (HEPA)-filtered air between tests to prevent carryover of aerosols (confirmed by a TSI optical particle counter) [18].

For the present study, we designed and built a new addition for the TARDIS. This was an Ultraviolet Germicidal Irradiation (UVGI) system, comprising 36 individual UV-C (265 nm) light-emitting diodes (LEDs) evenly spaced in a circular configuration across the diameter of the drum’s end plate. Industry-standard ray tracing software (Zemax OpticStudio, Zemax Extended Edition) was used to optimize the LED configuration light and distribution throughout the drum, which was validated empirically (see following section). The LEDs were fitted with a microprocessor control unit (to vary the output between 0 and 100% power) and a remote controller. The UV-C LEDs are covered by a metal plate, and all UV-C radiation was directed into, and contained within, the drum as confirmed by radiometer measurements (Model ILT 1400, International Light, Inc., Newburyport, MA, USA). The LEDs were near the peak disinfection wavelength for many organisms (265 nm) due to its strong absorption by the nucleic acid (compared with traditional lamps which are typically 254 nm) [27,28,50].

### 2.2. UV-C Validation

To validate the UV-C intensity inside the drum, we performed >300 individual radiometer measurements (with the drum closed and all UV-C LEDs at 100% power), spanning each combination of five positions along the axis (10, 35, 60, 85, 110 cm from the plate), three radial positions (0, 10, 20 cm), five angles to the axis (0, 45, 90, 135, 180°) and four rotations about the axis (0, 90, 180, 270°) to measure the UV-C incident on a sphere (i.e., to best approximate airborne *Pa*). We fitted a modified Akima spline in MATLAB to the measurements as an estimate of the mean and standard deviation of UV-C intensity incorporating all points in the drum. The overall mean UV-C intensity was 4.1 (SD: ±2.2) µW/cm^2^. This was scaled proportionately when the LEDs were at <100% power.

### 2.3. Relative Humidity and Temperature

The RH and temperature inside the drum were measured continuously by a thermo-hygrometer (HC2-C04, Rotronic Instrument Corp, Bassersdorf, Switzerland). Our primary focus was medium RH levels (target: 60 ± 5% at ambient temperature in the test laboratory), representative of an air-conditioned indoor healthcare setting. We did additional experiments to complement those at medium RH, spanning a realistic range of low (target: 40 ± 5%) through to high (target: 80 ± 5%) RH levels (described further in Section 2.11). RH levels were adjusted up and down by adding sterile water vapor or HEPA-filtered compressed air into the drum, respectively, until RH stabilised.

### 2.4. Culture Preparations

A representative isolate of the *Pa* AUST-02 (AUS023) strain, a dominant clinical strain in the Australian CF community [14], was used to prepare cultures (Supplement Page S1). AUST-02 was the main focus in this study. Other common Australian clinical strains of *Pa* derived from adults with CF attending the Prince Charles Hospital (TPCH), including a representative isolate of AUST-01 (ST649), AUST-06 (ST801) [14], and a representative non-clonal strain (ST155) were used to provide context to AUST-02 results, regarding potential strain-specific variation in UV-C effectiveness.

### 2.5. Nebulization

*Pa* was aerosolized using a Collison six-jet nebulizer connected to instrument-grade filtered compressed air (CN25; BGI Inc., Waltham, MA, USA). The aerosols were delivered into the drum at a rate of 16 L/min for ~10 s (a total of ~2.6–3 L).

### 2.6. Bioaerosol Sampling

Aerosols were collected using 6-stage Andersen Viable Cascade impactors (ACI, Thermo Fisher Scientific, Waltham, MA, USA), connected to a vacuum pump with a flow of 28.3 L/min for 5 min. The impactor has six size ranges (0.65 μm to >7 μm) as described previously [51]. Chocolate-bacitracin was used for the agar plates (prepared in-house). The air extracted by the impactor (and a TSI 9303 AeroTrak Optical Particle Counter [OPC]) was replaced with HEPA-filtered air [49], and each successive sample from the drum was equivalent to an air exchange rate of 0.36 AER of filtered air (i.e., an AER of 1.1 after extracting three samples). After finishing each test, the drum was flushed with HEPA-filtered air to purge residual aerosols through another HEPA filter and into a biosafety cabinet.

### 2.7. Enumeration of Airborne Pa

The inoculated agar plates used in the Andersen Cascade Impactor of all UV-C tests were covered completely with aluminum foil to negate potential for photoreactivation. All plates were incubated at 37 °C for 72 h. Plates were read for colony numbers at 24, 48, and 72 h using our previous protocols [17]. The total *Pa* colony counts were determined by summing all colonies at 72 h over impactor stages 1 to 6.

### 2.8. Particle Size Measurements

During experiments, the particle number concentration and size distribution of aerosols inside the drum were measured before and after impactor samples using a TSI Model 9303 AeroTrak OPC (TSI, Shoreview, MN, USA) at a flow rate of 2.83 L/min. There are six size channels with lower diameter cut-points from 0.3 to 10.0 μm.

### 2.9. Test Procedure

Test and cleaning procedures are further described in the Supplement (Pages S2–S3). Briefly, each test comprised a decay measurement with impactor samples extracted 5, 30, and 40 min after the *Pa* suspension was nebulized into the drum (termed Extraction A, B, and C, respectively). UV-C doses were delivered between Extraction A and B. Each testing day, one control (no UV-C) decay test was performed to account for day-to-day variation in *Pa* decay unrelated to UV-C (e.g., the *Pa* suspension, ambient conditions, and other factors that could influence the interpretation of UV-C results). The drum surface was swabbed and cultured at the start of testing days to assess for residual *Pa* and to confirm the effectiveness of cleaning performed after the prior study.

### 2.10. Core Experiments

A series of core experiments, each repeated ≥3 times, were implemented for a range of UV-C doses at 60% RH with the aim of spanning a range of effectiveness from the natural decay of *Pa* (i.e., 0% effective) through to 100% effective. The power of the UV-C LEDs were 1.25, 2.5, 5, 10, 20, 40 and 100% and were on for 20 min continuous exposure. The corresponding UV-C intensities were 0.05, 0.10, 0.21, 0.41, 0.82, 1.64, and 4.10 µW/cm^2^, respectively. The estimated doses delivered were therefore 62, 123, 246, 492, 984, 1968, and 4920 µW s/cm^2^, respectively.

### 2.11. Other Experiments

Additional experiments were performed for a subset of three doses (123, 492, and 1968 μW s/cm^2^) and control tests at lower (40%) RH and higher (80%) RH conditions. Each combination of dose and RH was repeated at least twice (Supplement Figure S1). The other sensitivity tests were performed once each for a subset of three doses (123, 492, and 1968 μW s/cm^2^), plus the no UV-C control condition, at 60% RH (Supplement Figure S1). The first set of experiments assessed the effect of delivering those doses over shorter (2 min) and longer (40 min) times. The purpose was to confirm if UV-C and *Pa* approximated the Bunsen-Roscoe reciprocity law (i.e., that the effects of a given dose are the same regardless of the time over which it is delivered [45]). The second set of experiments assessed the effect of UV-C on the other *Pa* isolates.

### 2.12. Statistical Analysis

Data were analyzed using SPSS version 27.0 (IBM Corp, Armonk, NY, USA). Colony forming unit (CFU) counts were corrected for coincidence error, using the methods described by Macher [52], due to the possibility of multiple viable *Pa* being deposited in a single impactor hole onto agar plates and being counted as a single CFU.

We based our estimates on the decay of airborne *Pa* over time, with and without UV-C, which is the standard method for decay-based bioaerosol experiments of UV-C [32,53]. First et al. [53] provide an extensive overview of the technical background to the derivation of basis of this metric. Briefly, without UV-C, the exponential decay in *Pa* over time was driven by natural decay due to factors other than UV-C (ventilation with filtered air as described above, and the biological decay of the organism [18]). When UV-C was operating, the total reduction in airborne *Pa* represented the combined effects of natural decay and additional UV-C inactivation. By comparing the total reduction with UV-C on and off, the difference in the slopes of decay (i.e., elapsed time in hours vs. natural logarithm of CFU count) was used to estimate the UV-C effectiveness as the equivalent air exchange rate (eAER) due to UV-C [53]. This is the amount of fresh air ventilation that would be required to yield the same removal as UV-C in a well-mixed space [53].

Additionally, we expressed the estimates of eAER as percentages (i.e., UV-C effectiveness, the percentage of total *Pa* inactivation that is attributable to UV-C), which are potentially more relevant to human infection risk assessment, as proposed by Ko et al. [54]. We present descriptive results using both methods, while the statistical analyses described below were based on percentages.

Continuous variables were summarized as mean and standard deviation (SD) if data were normally distributed or as median and interquartile range (IQR) if not. A Kruskal–Wallis test was used to examine natural decay across the three RH conditions at Extraction B and C. A one-way ANOVA was used to examine UV-C effectiveness across UV-C doses. A paired t-test was used to compare the total decay (i.e., due to both natural decay and UV-C) and natural decay. *p*-values < 0.05 were considered significant. As only a single test under each condition was conducted for isolates other than AUST-02, and exposure times other than 20 min, no formal comparisons were attempted.

A split plot ANOVA was performed to examine the effect of UV-C dose and RH on UV-C effectiveness where RH was considered a main effect. Estimated marginal means and standard errors were calculated.

### 2.13. Indoor Model

We used a basic model of upper air UV-C [55,56] to estimate what UV-C intensities are required in realistic outpatient clinical settings to deliver the doses we assessed, by combining our previous measurements of room geometry and ventilation rates (AER) in a tertiary hospital with a specialized adult CF service (Supplement Page S4) [57]. This included two outpatient consultation rooms and a large open-plan spirometry laboratory where patients congregate, all with standard mixing-type ventilation. The purpose was to place our empirical results in the context of what can practically and safely be delivered in occupied healthcare settings, drawing on the literature for tuberculosis (TB) control [55,58,59,60].

## 3. Results

### 3.1. General

In total, 120 experiments were performed, of which 112 (93%) were included in our analysis; eight experiments (7%) were excluded due to instrument failure. A single experiment took 3 h to complete, on average. The distribution of the experiments between different scenarios is shown in Supplement Figure S1.

The median temperature inside the drum was 21.1 °C (IQR: 19.5–22.3). For the low RH tests (target: 40%), the median measured RH inside the drum was 40.8% (IQR: 39.7–42.0). For medium RH tests (target: 60%), the median RH was 61.2% (IQR: 60.1–62.4). For high RH tests (target: 80%), the median RH was 80.9% (IQR: 80.4–81.9).

Almost all viable *Pa* ranged from 0.65 to 3.3 µm (stages 4–6 of Andersen impactor plates), with a median of 93% (IQR: 90.4–94.9) in that range. The predominance of *Pa* in that size range remained consistent between different experiments (Table S1), as was the case in the size distributions measured by the OPC (Figure S2). No residual *Pa* colonies were detected post-cleaning on the drum surface.

### 3.2. Natural Decay and Total Reduction in Airborne Pa

The median natural decay of airborne *Pa* at Extraction B was 56.5% (IQR: 52.1–57.6) at medium RH (*n* = 8). Results for other RH conditions were 44.8% (IQR: 42.4–50.9) at high RH (*n* = 11) and 83.5% (IQR: 83.3–85.3) at low RH (*n* = 3). Further information is in the Supplement (Figure S2).

Table S2 shows the total reduction in *Pa* at 60% RH across seven UV-C doses. It ranged from 57.0 (SD: 2.4) to 99.9 (SD: 0.04) when UV-C dose increased from 62 to 4920 µW s/cm^2^. Corresponding total eAER values were from 2.3 (SD: 0.2) to 17.9 (SD: 0.9) (Table S2).

### 3.3. UV-C Effectiveness

**Figure 2:**
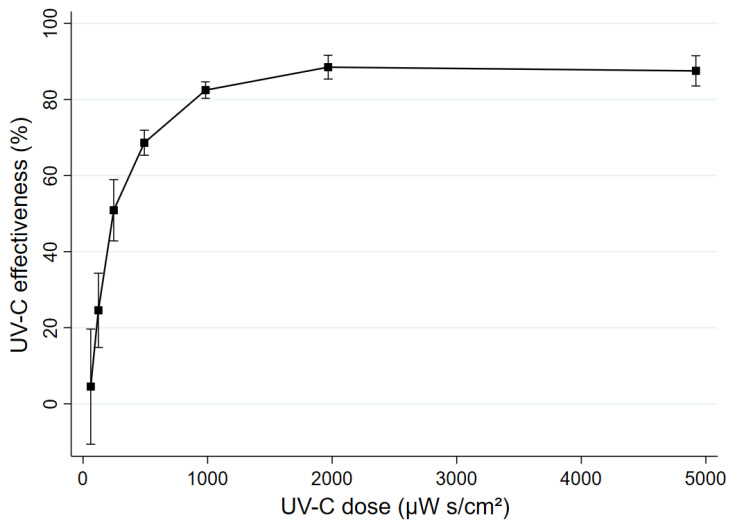
Mean UV-C effectiveness for airborne *Pa* AUST-02 at 60% RH by dose. Error bars are 95% confidence intervals.

Table 1 summarizes the estimated UV-C effectiveness at 60% RH. UV-C effectiveness ranged from 4.5% (SD: 6.1) to 88.5% (SD: 2.0) across the range of doses. Corresponding eAER values attributable to UV-C ranged from 0.1 (SD: 0.1) to 15.8 (SD: 1.1) (Table 1). Figure 2 shows the increase in UV-C effectiveness across UV-C doses. ANOVA suggested that the mean effectiveness of UV-C doses between 62 and 492 µW s/cm^2^ were significantly different from each other, and compared with higher doses (>492 µW s/cm^2^) (Table S3). In contrast, the effectiveness of doses ≥984 µW s/cm^2^ were not significantly different from each other (*p*-values: 0.365 to ~1), as is highlighted in the flattening of the line shown in Figure 2.

At 60% UV-C significantly (*p* < 0.005) increased the total inactivation of *Pa* compared with natural decay at all doses except 62 µW s/cm^2^ (Table S4).

### 3.4. Other Experiments

The effectiveness of UV-C doses showed some dependence on RH levels although the overall results were mixed (Table S5). We observed little difference in UV-C effectiveness over the range of UV-C doses examined between medium and high RH. At low RH, we observed differences in effectiveness compared to high RH only at the lowest and highest doses of UV-C tested (123 and 1968 µW s/cm^2^), although the direction of the difference was not consistent. Detailed results are in the Supplement (Page S7).

UV-C effectiveness at 60% RH for other different *Pa* isolates (AUST-01, AUST-02, AUST-06 and the non-clonal strain) suggested some variability compared with that observed for AUST-02, which was more apparent at lower doses (Table S6). Delivering the same UV-C dose over 20 min or 40 min led to comparable results, which did not suggest a departure from the Bunsen-Roscoe law (Table S7). Delivering the UV-C doses over 2 min led to higher effectiveness estimates, which could be an artefact of low numbers of viable *Pa* colonies in those experiments and the use of a single test.

### 3.5. Indoor Model Results

The estimated exposure times in the upper air irradiated zone (the upper 0.675 m of three clinical rooms with ceiling of 2.7 m) of *Pa* droplet nuclei, were 1.2, 1.4 and 1.6 min, respectively, (Supplement Table S8). To achieve the dose above which higher doses did not significantly increase effectiveness (984 µW s/cm^2^), which was 83% effective at inactivating airborne *Pa*, would require average UV-C intensities of 14, 11 and 10 µW/cm^2^ in the upper room, respectively (Supplement Table S9).

For a standard UV-C installation (i.e., louvered wall-mounted UV-C fixture that irradiates the upper ~0.7 m of room), CDC/NIOSH guidelines report measured mean eye-level (1.5 m) intensity is 0.46% of that in the upper air. Applying that to the required intensities in the clinical rooms we modelled yielded mean estimated eye-level intensities of 0.063, 0.054 and 0.047 µW/cm^2^ in the lower room, which are 49%, 42%, and 37%, respectively, of the occupational limit for 265 nm UV-C over 8 h in any 24 h period (37 J/m^2^) [61]. The equivalent figures for the doses at which approximately 50% and 90% of *Pa* were inactivated by UV-C (246 and 1968 µW s/cm^2^) ranged from 12–98%, 10–84% and 9–73%, respectively, in the three rooms (Supplement Table S10). Estimates for all doses are presented in the Supplement (Table S10).

## 4. Discussion

We quantified the effectiveness of UV-C to inactivate or kill aerosolized *Pa*, which can be transmitted by the airborne route in people with CF. We observed that UV-C doses from 246–1968 µW s/cm^2^ had an estimated effectiveness of approximately 50–90% for airborne *Pa* at 60% RH. The addition of UV-C significantly increased the inactivation of *Pa* compared with natural decay alone at all but one of the doses assessed. Consistent with UV-C theory [56], inactivation was approximately a first-order process that increases before reaching a plateau. For example, UV-C effectiveness was not significantly different when UV-C doses exceeded 984 μW s/cm^2^.

### 4.1. Comparison with Other Studies

The results of previous studies [34,45,46] are not directly comparable to ours due to methodological differences (e.g., experimental set-up, test conditions and how outcome measures were defined). We used UV-C at 265 nm, while previous studies used 254 nm [34]. Specifically, 265 nm is closer to the theoretical peak germicidal wavelength for most organisms [62,63,64,65]. Another factor which makes it challenging to compare results is that we used a clinical strain of *Pa* (AUST-02), rather than the commercially available laboratory strain (ATCC 27853) used in a previous study [34]. It is not possible to state how much those factors, or indeed others, contribute individually or in combination.

Noting the issues outlined above, some key findings are summarized below. One study reported that UV-C doses of 1000 µW s/cm^2^ were required for ~90% inactivation at 43% RH [46]. Another study found that at medium RH (58.7–59.6%) [34], UV-C doses of approximately 3289 to 20,933 µW s/cm^2^ were required for 90% inactivation. In the earliest study identified, the UV dose (of which >88% was 254 nm UV-C) required to achieve ~99.99% reduction in airborne *Pa* was ~1600 µW s/cm^2^, although that was measured at an RH of 95% [45].

Based on the limited empirical data available, airborne *Pa* seem to fall in the middle of the susceptibility spectrum among airborne bacteria treated with UV-C. Similar to *M. tuberculosis*, *Pa* appear to be neither highly sensitive to UV-C’s germicidal effects like *Streptococcus mitis*, nor highly resistant like *Bacillus subtilis* and other spore-forming organisms [46,55,60]. The pronounced heterogeneity in susceptibility supports the need to assess bacteria on a case-by-case basis, as generalizability may be limited [34,46]. The same issue of generalizability applies to assessing a given organism across a range of indoor environmental conditions, as survival is complex, highly species-dependent, even among Gram-negative bacteria [34,35,36,66,67].

### 4.2. Relevance to Infection Control in People with CF

Despite first being applied to airborne pathogen control in the 1930s, the application of UV-C beyond laboratories and some indoor health care settings has been more sporadic than might be expected [26] given its performance in those early studies. Most studies during the latter part of the 20th century focused on TB using organisms including *Mycobacterium bovis* and BCG, and UV-C had a resurgence during the 1980s driven by the unexpected rise in TB notification in North America at that time [55] in susceptible individuals.

Outcomes of the important historical research are relevant to planning if and where to use UV-C for *Pa* control in CF centres. Specifically, that UV-C is generally best suited to applications in upper air of occupied rooms in congregate settings, because this is closest to the source of *Pa* and is where susceptible people [56] are located. That is why UV-C treatment of air in ventilation ducts is not generally recommended for TB control, because most transmission occurs in room rather than through HVAC systems [59]. Similarly, applying very high UV-C intensity from ‘bare bulb’ fixtures in unoccupied rooms, following the departure of the infectious source, has the theoretical benefit of treating air and surfaces. Despite that, it is not recommended for TB control [59] because it cannot treat the air at the time of greatest risk when the source is in the room and producing droplet nuclei. The effectiveness of such approaches for person-to-person *Pa* pathogen nuclei is unclear.

*Pa* is not exclusively transmitted by the airborne route like TB, so any implementation of UV-C needs to consider that important distinction. In locations within CF centers where person-to-person transmission of *Pa* is the primary concern, upper-room UV-C in occupied rooms appears to be a higher priority than other UV-C implementations, but further research is needed to confirm the relative benefits of these. Overall, for *Pa* a multi-faceted approach in settings where people with CF congregate including airborne (upper-room UV-C, adequate outdoor air ventilation) and large droplet (surgical masks [24], social distancing) and surface cleaning controls seems prudent [59].

### 4.3. Safety and Implementation

Our modeling of empirical ventilation and geometry observations in three clinical rooms at a functioning CF Centre estimated that an upper-air dose required for 83% *Pa* inactivation would correspond to lower room doses that were 37–49% of the 8 h in occupational limit for 265 nm UV-C. For ~90% inactivation, the equivalent estimates doses ranged from 73–98% of the daily limit.

UV-C doses appear to be safe to implement in indoor settings, although we caution those estimates require validation with radiometer measurements in clinical rooms. There are several other factors that require consideration. For example, our estimates assume continuous eye exposure to UV-C for 8 h, known to be highly unlikely in practice [58]. Measured personal exposures of healthcare workers and inpatients demonstrates doses between 0.5% and 8.8% of those calculated assuming continuous exposure, even for inpatients who rarely leave their room (e.g., while undergoing TB isolation [68]). Applying 8.8% to our estimates, for the scenario targeting 83% *Pa* inactivation, corresponds to 3.3%–4.3% of the 8 h limit received over that time by occupants of the lower room. The equivalent figures for the UV-C dose at which ~90% of *Pa* was inactivated were 6.4%–8.6% of the 8 h limit.

Patient and staff safety is paramount in occupied settings. Appropriately installed and maintained upper-room UV-C fixtures are safe [59], posing no risk of photokeratitis or skin erythema, as shown in the largest study of 3611 subjects in a double-blind placebo-controlled field study over eight years [58]. The authors of that study also conducted a literature review, identifying only five published case reports of UV-C overexposure, all of which were due to human error, and only one instance involved a properly installed UV-C fixture [58]. In that case, the error was that the standard practice of performing measurements after commissioning a new fixture was not followed.

Even in situations where UV-C doses exceeded occupational limits by 20–100 times, ocular symptoms resolved within two to four days, and skin erythema symptoms resolved within two weeks, with no complications. UV-C does not the penetrate skin cells as efficiently as longer wavelength UV-B or UV-A band light, while the cornea is more sensitive because it has no outer layer of dead cells to attenuate UV, although UV-C does not penetrate to the lens or retina [55]. Photokeratitis can be painful but resolves without sequalae [58] in 48 h once the source of the high exposure has been removed [58]. In sum, the germicidal benefits of UV-C for preventing or limiting infection among susceptible individuals, like *Pa* transmission in people with CF in outpatient or inpatient settings, exceed the negligible risks posed [59].

### 4.4. Limitations

Important limitations of our study include: (1) The infectious inoculum for *Pa* is unknown, and like other studies it is not possible to place our results in the context of preventing *Pa* infection. (2) We used a clinical strain suspended in a 10% fetal bovine serum (FBS) and phosphate-buffered saline (PBS) solution. We have recently shown these mixtures showed similar physical and chemical properties to cough aerosol from healthy adults, including hygroscopicity [69,70,71,72]. However, the extent to which that is generalizable to *Pa* droplet nuclei from generated by people with CF is unknown. (3) UV-C intensities along the length of the drum were not uniform. This is analogous to the common configuration of having an upper-room UV-C lamp on one side of a small room. We therefore used measurements to estimate an empirical intensity in the drum, and its uncertainty at different power levels, which agreed well with theoretical model-based estimates that informed the drum design. (4) We had a limited number (three) of time points at which we measured the decay of *Pa* over 45 min. We repeated each test in the main series of experiments 3–4 times, as and our previous work found this was an adequate combination for measuring the natural decay of *Pa* [18]. (5) We conducted fewer experiments for a subset of doses for the low and high RH conditions, non-AUST-02 *Pa* strains, and also when UV-C was on for 2 min and 40 min. Those experiments were done to provide context for the main experiments and should be interpreted cautiously.

## 5. Conclusions

UV-C can expedite inactivation of nebulized airborne *Pa* droplet nuclei of the most common clonal Pa strain found in Australians with CF. This was observed at UV-C doses that modelling suggests can be implemented safely in occupied indoor settings. These findings could be corroborated in vivo in cough aerosol from people with CF in future. UV-C may have a role as part of a multi-faceted infection control strategy that incorporates other indoor, individual and administrative control measures in clinical settings where people with CF congregate.

## Figures and Tables

**Table 1:** UV-C effectiveness (%) against airborne *Pa* AUST-02 at 60% RH and their equivalent air exchange rates (eAER) per hour.

UV-C Dose (µW s/cm^2^)	Total *n* = 34	UV-C Effectiveness (%, SD)	Equivalent Air Exchange Rate due to UV-C (eAER, SD)
0 *	8	55.2 (4.4) ^§^	2.2 (0.2) ^†^
62	3	4.5 (6.1)	0.1 (0.1)
123	4	24.6 (6.1)	0.7 (0.2)
246	4	50.9 (5.1)	2.3 (0.3)
492	4	68.6 (2.1)	4.5 (0.8)
984	4	82.5 (1.4)	10.0 (1.7)
1968	4	88.5 (2.0)	15.8 (1.1)
4920	3	87.5 (1.6)	15.6 (0.8)

Note: * no UV-C (control) test of natural decay is denoted as 0 dose, ^§^ mean natural decay when UV-C was off, ^†^ this is the non-UVC AER due to ventilation and natural decay only.

## Data Availability

Not applicable.

## Supplementary Materials

The following supporting information can be downloaded at: https://www.mdpi.com/article/10.3390/ijerph192013706/s1, Figure S1: Outline of the experiments; Figure S2: Box plots of the natural decay (%) of aerosolized *Pa* AUST-02 by extraction time and RH; Figure S3: Particle concentrations and size distribution inside the drum at medium RH; Table S1: Viable *Pa* (as a % of all size ranges) in the size range from 0.65 to 3.3. µm captured by ACI stages 4-6; Table S2: Mean total decay (%) of airborne *Pa* AUST-02 at 60% RH and their equivalent air exchange rates (eAER) per hour; Table S3: ANOVA for the difference in UV-C effectiveness (%) between different doses of UV-C against airborne *Pa* AUST-02 at medium RH; Table S4: Paired-samples t-test for the difference in the mean total decay (%) (I) and natural decay (J) of airborne *Pa* AUST-02 at medium RH; Table S5: Split plot ANOVA for the effects of RH and UV-C dose on UV-C effectiveness (%); Table S6: UV-C effectiveness (%) against different genotypes of *Pa* at medium RH; Table S7: UV-C effectiveness (%) against airborne *Pa* AUST-02 at different UV-C exposure times at medium RH; Table S8: Basic information of the rooms at TPCH and the assumptions for upper-room UVGI air disinfection estimation; Table S9: The upper room exposure time and the minimum average UV-C intensity required that correspond to the experimental results of total reduction of airborne *Pa* AUST-02; Table S10: The eye level estimated UV-C intensity required that correspond to the experimental results of total reduction of airborne *Pa* AUST-02.

## References

[1] O’Sullivan B.P., Freedman S.D. (2009). Cystic fibrosis. Lancet.

[2] Gaspar M.C., Couet W., Olivier J.C., Pais A.A., Sousa J.J. (2013). Pseudomonas aeruginosa infection in cystic fibrosis lung disease and new perspectives of treatment: A review. Eur. J. Clin. Microbiol. Infect. Dis..

[3] Mahenthiralingam E. (2014). Emerging cystic fibrosis pathogens and the microbiome. Paediatr. Respir. Rev..

[4] Elborn J.S. (2008). Identification and management of unusual pathogens in cystic fibrosis. J. R Soc. Med..

[5] Dodge J.A., Lewis P.A., Stanton M., Wilsher J. (2007). Cystic fibrosis mortality and survival in the UK: 1947–003. Eur. Respir. J..

[6] Keogh R.H., Szczesniak R., Taylor-Robinson D., Bilton D. (2018). Up-to-date and projected estimates of survival for people with cystic fibrosis using baseline characteristics: A longitudinal study using UK patient registry data. J. Cyst. Fibros..

[7] Ahern S., Caruso M., Ruseckaite R., BobotA S.B.N. (2021). The Australian Cystic Fibrosis Data Registry Annual Report 2020.

[8] Rachel M., Topolewicz S., Śliwczyński A., Galiniak S. (2020). Managing Cystic Fibrosis in Polish Healthcare. Int. J. Environ. Res. Public Health.

[9] CF Australia (2007). Infection Control Guidelines for Cystic Fibrosis Patients and Carers.

[10] Suarez C., Pena C., Arch O., Dominguez M.A., Tubau F., Juan C., Gavalda L., Sora M., Oliver A., Pujol M. (2011). A large sustained endemic outbreak of multiresistant Pseudomonas aeruginosa: A new epidemiological scenario for nosocomial acquisition. BMC Infect. Dis..

[11] Saiman L., Siegel J., Cystic Fibrosis F. (2003). Infection control recommendations for patients with cystic fibrosis: Microbiology, important pathogens, and infection control practices to prevent patient-to-patient transmission. Infect. Control. Hosp. Epidemiol..

[12] Bryant J.M., Grogono D.M., Greaves D., Foweraker J., Roddick I., Inns T., Reacher M., Haworth C.S., Curran M.D., Harris S.R. (2013). Whole-genome sequencing to identify transmission of Mycobacterium abscessus between patients with cystic fibrosis: A retrospective cohort study. Lancet.

[13] Hansen C.R., Pressler T., Ridderberg W., Johansen H.K., Skov M. (2013). Achromobacter species in cystic fibrosis: Cross-infection caused by indirect patient-to-patient contact. J. Cyst. Fibros..

[14] Kidd T.J., Ramsay K.A., Hu H., Marks G.B., Wainwright C.E., Bye P.T., Elkins M.R., Robinson P.J., Rose B.R., Wilson J.W. (2013). Shared Pseudomonas aeruginosa genotypes are common in Australian cystic fibrosis centres. Eur. Respir. J..

[15] Clifton I.J., Peckham D.G. (2010). Defining routes of airborne transmission of Pseudomonas aeruginosa in people with cystic fibrosis. Expert. Rev. Respir. Med..

[16] Schaffer K. (2015). Epidemiology of infection and current guidelines for infection prevention in cystic fibrosis patients. J. Hosp. Infect..

[17] Wainwright C.E., France M.W., O’Rourke P., Anuj S., Kidd T.J., Nissen M.D., Sloots T.P., Coulter C., Ristovski Z., Hargreaves M. (2009). Cough-generated aerosols of Pseudomonas aeruginosa and other Gram-negative bacteria from patients with cystic fibrosis. Thorax.

[18] Knibbs L.D., Johnson G.R., Kidd T.J., Cheney J., Grimwood K., Kattenbelt J.A., O’Rourke P.K., Ramsay K.A., Sly P.D., Wainwright C.E. (2014). Viability of Pseudomonas aeruginosa in cough aerosols generated by persons with cystic fibrosis. Thorax.

[19] Wood M.E., Stockwell R.E., Johnson G.R., Ramsay K.A., Sherrard L.J., Kidd T.J., Cheney J., Ballard E.L., O’Rourke P., Jabbour N. (2019). Cystic fibrosis pathogens survive for extended periods within cough-generated droplet nuclei. Thorax.

[20] Bryant J.M., Grogono D.M., Rodriguez-Rincon D., Everall I., Brown K.P., Moreno P., Verma D., Hill E., Drijkoningen J., Gilligan P. (2016). Emergence and spread of a human-transmissible multidrug-resistant nontuberculous mycobacterium. Science.

[21] Saiman L., Siegel J.D., LiPuma J.J., Brown R.F., Bryson E.A., Chambers M.J., Downer V.S., Fliege J., Hazle L.A., Jain M. (2014). Infection prevention and control guideline for cystic fibrosis: 2013 update. Infect. Control. Hosp. Epidemiol..

[22] Harrison J., Pickering C.A.C., Faragher E.B., Austwick P.K.C., Little S.A., Lawton L. (1992). An Investigation of the Relationship between Microbial and Particulate Indoor Air-Pollution and the Sick Building Syndrome. Resp. Med..

[23] Vanden Driessche K., Hens N., Tilley P., Quon B.S., Chilvers M.A., de Groot R., Cotton M.F., Marais B.J., Speert D.P., Zlosnik J.E. (2015). Surgical masks reduce airborne spread of Pseudomonas aeruginosa in colonized patients with cystic fibrosis. Am. J. Respir. Crit. Care Med..

[24] Wood M.E., Stockwell R.E., Johnson G.R., Ramsay K.A., Sherrard L.J., Jabbour N., Ballard E., O’Rourke P., Kidd T.J., Wainwright C.E. (2018). Face Masks and Cough Etiquette Reduce the Cough Aerosol Concentration of Pseudomonas aeruginosa in People with Cystic Fibrosis. Am. J. Respir. Crit. Care Med..

[25] Stockwell R.E., Wood M.E., He C., Sherrard L.J., Ballard E.L., Kidd T.J., Johnson G.R., Knibbs L.D., Morawska L., Bell S.C. (2018). Face Masks Reduce the Release of Pseudomonas aeruginosa Cough Aerosols When Worn for Clinically Relevant Periods. Am. J. Respir. Crit. Care Med..

[26] Reed N.G. (2010). The history of ultraviolet germicidal irradiation for air disinfection. Public Health Rep..

[27] Dai T., Vrahas M.S., Murray C.K., Hamblin M.R. (2012). Ultraviolet C irradiation: An alternative antimicrobial approach to localized infections?. Expert Rev. Anti. Infect. Ther..

[28] Guerrero-Beltr·n J.A., Barbosa-C·novas G.V. (2004). Advantages and Limitations on Processing Foods by UV Light. Food Sci. Technol. Int..

[29] Wells W.F., Fair G.M. (1935). Viability of B. Coli Exposed to Ultra-Violet Radiation in Air. Science.

[30] King B., Kesavan J., Sagripanti J.L. (2011). Germicidal UV Sensitivity of Bacteria in Aerosols and on Contaminated Surfaces. Aerosol. Sci. Technol..

[31] Kethley T.W., Branch K. (1972). Ultraviolet lamps for room air disinfection. Effect of sampling location and particle size of bacterial aerosol. Arch. Environ. Health.

[32] Riley R.L., Knight M., Middlebrook G. (1976). Ultraviolet susceptibility of BCG and virulent tubercle bacilli. Am. Rev. Respir Dis.

[33] Peccia J., Hernandez M. (2004). UV-induced inactivation rates for airborne Mycobacterium bovis BCG. J. Occup. Environ. Hyg..

[34] Chang C.W., Li S.Y., Huang S.H., Huang C.K., Chen Y.Y., Chen C.C. (2013). Effects of ultraviolet germicidal irradiation and swirling motion on airborne Staphylococcus aureus, Pseudomonas aeruginosa and Legionella pneumophila under various relative humidities. Indoor Air.

[35] Ko G., First M.W., Burge H.A. (2000). Influence of relative humidity on particle size and UV sensitivity of Serratia marcescens and Mycobacterium bovis BCG aerosols. Tuber. Lung Dis..

[36] Peccia J., Werth H.M., Miller S., Hernandez M. (2001). Effects of relative humidity on the ultraviolet induced inactivation of airborne bacteria. Aerosol. Sci. Technol..

[37] Baldelli G., Aliano M.P., Amagliani G., Magnani M., Brandi G., Pennino C., Schiavano G.F. (2022). Airborne Microorganism Inactivation by a UV-C LED and Ionizer-Based Continuous Sanitation Air (CSA) System in Train Environments. Int. J. Environ. Res. Public Health.

[38] Viana Martins C.P., Xavier C.S.F., Cobrado L. (2022). Disinfection methods against SARS-CoV-2: A systematic review. J Hosp Infect..

[39] Qiao Y., Yang M., Marabella I.A., McGee D.A.J., Aboubakr H., Goyal S., Hogan C.J., Olson B.A., Torremorell M. (2021). Greater than 3-Log Reduction in Viable Coronavirus Aerosol Concentration in Ducted Ultraviolet-C (UV-C) Systems. Environ. Sci. Technol..

[40] Ueki H., Ito M., Furusawa Y., Yamayoshi S., Inoue S.I., Kawaoka Y. (2022). A 265-Nanometer High-Power Deep-UV Light-Emitting Diode Rapidly Inactivates SARS-CoV-2 Aerosols. mSphere.

[41] VanOsdell D., Foarde K. (2002). Defining the Effectiveness of UV Lamps Installed in Circulating Air Ductwork.

[42] Rudnick S.N., First M.W. (2007). Fundamental Factors Affecting Upper-Room Ultraviolet Germicidal IrradiationÔÇöPart II. Predicting Effectiveness. J. Occup. Environ. Hyg..

[43] Li P.Y., Li L., Wang Y.J., Zheng T.L., Liu J.X. (2021). Characterization, factors, and UV reduction of airborne bacteria in a rural wastewater treatment station. Sci. Total Environ..

[44] Nguyen T.T., Johnson G.R., Bell S.C., Knibbs L.D. (2022). A Systematic Literature Review of Indoor Air Disinfection Techniques for Airborne Bacterial Respiratory Pathogens. Int. J. Environ. Res. Public Health.

[45] Sharp D.G. (1940). The effects of ultraviolet light on bacteria suspended in air. J. Bacteriol..

[46] Noakes C.J., Fletcher L.A., Beggs C.B., Sleigh P.A., Kerr K.G. (2004). Development of a numerical model to simulate the biological inactivation of airborne microorganisms in the presence of ultraviolet light. J. Aerosol Sci..

[47] Beggs C.B., Kerr K.G., Donnelly J.K., Sleigh P.A., Mara D.D., Cairns G. (2000). The resurgence of tuberculosis in the tropics. An engineering approach to the control of Mycobacterium tuberculosis and other airborne pathogens: A UK hospital based pilot study. Trans. R Soc. Trop. Med. Hyg..

[48] Collins F.M. (1971). Relative susceptibility of acid-fast and non-acid-fast bacteria to ultraviolet light. Appl. Microbiol..

[49] Johnson G.R., Knibbs L.D., Kidd T.J., Wainwright C.E., Wood M.E., Ramsay K.A., Bell S.C., Morawska L. (2016). A Novel Method and Its Application to Measuring Pathogen Decay in Bioaerosols from Patients with Respiratory Disease. PLoS ONE.

[50] Sónia Gonçalves Pereira I.P.M., Ana Cristina Rosa and Olga Cardoso (2015). Susceptibility to ultraviolet light C of Pseudomonas aeruginosa biofilms from hydropathic respiratory treatment equipments: Impact in water quality control and public health. Int. J. Curr. Microbiol. Appl. Sci..

[51] Andersen A.A. (1958). New sampler for the collection, sizing, and enumeration of viable airborne particles. J. Bacteriol..

[52] Macher J.M. (1989). Positive-hole correction of multiple-jet impactors for collecting viable microorganisms. Am. Ind. Hyg. Assoc. J..

[53] First M., Rudnick S.N., Banahan K.F., Vincent R.L., Brickner P.W. (2007). Fundamental factors affecting upper-room ultraviolet germicidal irradiation—part I. Experimental. J. Occup. Environ. Hyg..

[54] Ko G., First M.W., Burge H.A. (2002). The characterization of upper-room ultraviolet germicidal irradiation in inactivating airbone microorganisms. Environ. Health Perspect..

[55] Brickner P.W., Vincent R.L., First M., Nardell E., Murray M., Kaufman W. (2003). The application of ultraviolet germicidal irradiation to control transmission of airborne disease: Bioterrorism countermeasure. Public Health Rep..

[56] First MW N.E., Chaisson W., Riley R. (1999). Guidelines for the application of upper-room ultraviolet germicidal irradiation for preventing transmission of airborne contagion-Part I: Basic principles. Trans. Am. Soc. Heat. Refrig. Air Cond. Eng..

[57] Knibbs L.D., Morawska L., Bell S.C., Grzybowski P. (2011). Room ventilation and the risk of airborne infection transmission in 3 health care settings within a large teaching hospital. Am. J. Infect. Control..

[58] Nardell E.A., Bucher S.J., Brickner P.W., Wang C., Vincent R.L., Becan-McBride K., James M.A., Michael M., Wright J.D. (2008). Safety of upper-room ultraviolet germicidal air disinfection for room occupants: Results from the Tuberculosis Ultraviolet Shelter Study. Public Health Rep..

[59] Nardell E., Vincent R., Sliney D.H. (2013). Upper-room ultraviolet germicidal irradiation (UVGI) for air disinfection: A symposium in print. Photochem. Photobiol..

[60] NIOSH (2009). Environmental Control for Tuberculosis: Basic Upper-Room Ultraviolet Germicidal Irradiation Guidelines for Healthcare Settings.

[61] Agency ARPaNS (2006). Radiation Protection Series No. 12. Radiation Protection Standard, Occupational Exposure to Ultraviolet Radiation.

[62] Bergman R.S. (2021). Germicidal UV Sources and Systems(dagger). Photochem. Photobiol..

[63] Beck S.E., Wright H.B., Hargy T.M., Larason T.C., Linden K.G. (2015). Action spectra for validation of pathogen disinfection in medium-pressure ultraviolet (UV) systems. Water Res..

[64] Kim S.J., Kim D.K., Kang D.H. (2016). Using UVC Light-Emitting Diodes at Wavelengths of 266 to 279 Nanometers To Inactivate Foodborne Pathogens and Pasteurize Sliced Cheese. Appl. Environ. Microb..

[65] Chen R.Z., Craik S.A., Bolton J.R. (2009). Comparison of the action spectra and relative DNA absorbance spectra of microorganisms: Information important for the determination of germicidal fluence (UV dose) in an ultraviolet disinfection of water. Water Res..

[66] Tang J.W. (2009). The effect of environmental parameters on the survival of airborne infectious agents. J. R Soc. Interface.

[67] Lin C.Y., Li C.S. (2002). Control effectiveness of ultraviolet germicidal irradiation on bioaerosols. Aerosol. Sci. Technol..

[68] First M.W., Weker R.A., Yasui S., Nardell E.A. (2005). Monitoring human exposures to upper-room germicidal ultraviolet irradiation. J. Occup. Environ. Hyg..

[69] Clifton I.J., Fletcher L.A., Beggs C.B., Denton M., Peckham D.G. (2008). A laminar flow model of aerosol survival of epidemic and non-epidemic strains of Pseudomonas aeruginosa isolated from people with cystic fibrosis. BMC Microbiol..

[70] Jones R.M., Brosseau L.M. (2015). Aerosol transmission of infectious disease. J. Occup. Environ. Med..

[71] Groth R., Cravigan L.T., Niazi S., Ristovski Z., Johnson G.R. (2021). In situ measurements of human cough aerosol hygroscopicity. J. R. Soc. Interface.

[72] Niazi S., Groth R., Cravigan L., He C.R., Tang J.W., Spann K., Johnson G.R. (2021). Susceptibility of an Airborne Common Cold Virus to Relative Humidity. Environ. Sci. Technol..

