# Supplementary material for: The Effectiveness of Ultraviolet-C (UV-C) Irradiation on the Viability of Airborne Pseudomonas aeruginosa
Source: Int J Environ Res Public Health. 2022 Oct 21;19(20):13706. doi: 10.3390/ijerph192013706 (PMC9602727; doi:10.3390/ijerph192013706)
Supplement: Supplementary file 1 [file ijerph-19-13706-s001.zip › ijerph-1958940-supplementary.pdf]

The effectiveness of ultraviolet-C (UV-C) irradiation on the viability of airborne *Pseudomonas aeruginosa*

Supplementary file

Culture preparation

A single colony of *Pa* AUST-02 (AUS023) strain, a dominant clinical strain in the Australian CF community [1], was inoculated into 20 ml of sterile lysogeny broth (LB) and incubated overnight at 37°C at 220 rpm. The overnight culture was pelleted at 4500 rpm for 10 min and the supernatant removed. The pellet was then resuspended in 20 ml of sterile 1X phosphate-buffered saline (PBS), and the washed culture was centrifuged at 4500 rpm for 10 min. After removing the supernatant, the pellet was resuspended in 15 ml of sterile 1X PBS. A spectrophotometer (BioPhotometer model 6131, Eppendorf) was used to adjust the bacterial concentration (with sterile 1X PBS) to an OD_{600} of 1.0 (i.e., approximately 4×10^8 cfu/ml). This suspension was then diluted to a concentration of approximately 4×10^5 cfu/ml for the next steps.

Next, 6 ml of sterile fetal bovine serum (FBS) was added to 39 ml of sterile 1X PBS in a Class-II Biological safety cabinet. Then, 15 ml of the 4×10^5 cfu/ml bacterial suspension was added to the FBS-PBS solution (i.e., a 1/4 dilution to give a bacterial concentration of ~1×10^5 cfu/ml in 10% FBS). Ten ml volumes of the solution were aliquoted into 6 × 15 ml sterile Falcon tubes and refrigerated. Each tube of the suspension was brought to room temperature (approximately 5 min on bench) just prior to each nebulization procedure. For microbiological control, 10 μL of the suspension were inoculated on a chocolate-bacitracin agar plate and incubated at 37°C for 3 days for the colony forming unit (CFU) count.

The suspension preparation procedure was followed for other common Australian clinical strains of *Pa* derived from adults with CF attending TPCH including a representative isolate of AUST-01 (ST649), AUST-06 (ST801) [1], and a representative non-clonal strain (ST155).

Test procedure

The test procedure was refined following pilot tests of the *Pa* suspension concentration, sample aging intervals, and Andersen impactor extraction times, which sought sufficient initial *Pa* to register change over the time period required to deliver different UV-C doses, but without saturating the CFU concentration on the plates preventing *Pa* enumeration.

First, 10 ml of *Pa* suspension was into the drum for 10 s, after which it was aged for 5 min to allow for mixing and for ambient conditions in the drum to stabilize. During the aging stage, the drum was rotated at 1.7 rpm to minimize gravitational settling and the inertial impaction of particles, following our previous methods [2]. Second, Andersen impactor samples were collected for 5 min (extraction A), after which the total elapsed time was 10 min, to determine the initial concentration of airborne *Pa* prior to treatment. Next, the aerosols were aged for a further 20 min with either UV-C off (i.e., control condition to establish the natural decay rate of *Pa* in the drum) or UV-C (to determine any additional decay due to UV-C). Third, a second 5-min Andersen impactor sample was collected (extraction B), after which the elapsed time was 35 min. Fourth, the aerosols were aged for another 5 min, before a third and final Andersen impactor sample was collected (extraction C), after which the elapsed time was 45 min.

New tests were only commenced after the OPC recorded a concentration of 0 in the 0.3 μm size channel.

Cleaning

After finishing each test, the nebulizer was cleaned by laboratory grade demineralized water 3-4 times and then dried by compressed air. Andersen impactors and associated fittings were sprayed with 80% ethanol at least 10 min prior to wipe out using all-purpose tissue. The drum was flushed with HEPA-filtered air to purge the remaining aerosols through a HEPA filter to biosafety cabinet. Once the OPC confirmed the concentration of 0 in the 0.3 μm, the new test was commenced.

At the end of each day, the nebulizer was treated with bleach (10% v/v) for a contact time of at least 20 min, then washed with laboratory grade demineralized water and dried by filtered compressed air. Andersen impactors and associated fittings were sprayed with 80% ethanol at least 10 min prior to air drying.

The drum was flushed with HEPA-filtered air to purge the remaining aerosols through a safety cabinet. Once the OPC confirmed the concentration was 0, the drum was opened for cleaning. Benzalkinium chloride (Glitz) (0.15% v/v) was used to clean inside the drum. This step was repeated twice. Following that, 80% ethanol was sprayed into the drum’s surfaces, and the valves and the connectors allowed to dry. The ethanol step was also repeated twice.

Upper-room Ultraviolet germicidal irradiation (UVGI) model

Based on the experimental data, a basic model of upper-room UVGI air disinfection was produced to provide indicative estimates of what UV-C intensities are required in three realistic clinical settings (spirometry laboratory and two outpatient consultation rooms) by combining our previous measurements of room geometry and ventilation rates at TPCH [3]. Briefly, the room height is 2.7 m and the total ACH of the lung function room and consultant room A and B were 8.5, 7 and 6.1, respectively, and all rooms had standard mixing ventilation configurations. For this simple calculation, we followed the assumptions of Brickner and colleagues [4]: (1) the room is divided into to two equal volumes and the lower 1.35 m of air circulate vertically through the upper 1.35 m; (2) the average height from which the lower room air rises was 0.675 m above the floor; (3) an amount of air equal to twice the lower ACH circulates through the upper room each hour; (4) UV-C lamp was installed at the middle of the upper room and UV-C irradiation is the upper 0.675 m only. As air circulates up and down, airborne *Pa* is in the irradiated zone for a period of time defined by the air velocity, and the target UV-C dose can be divided by that time in the irradiated zone to indicate the average UV-C intensity required across the zone [4].

Results

The ACH of three clinical rooms we previously measured (all with standard mixing ventilation configurations) were 8.5, 7.0 and 6.1, respectively, which equates to the air cycling (up and down) approximately 17, 14, and 12 times every hour (distance of ~34, ~28 and ~25 m, respectively). The air velocity in these rooms was 0.6, 0.5, and 0.4 m/min, respectively.

Table S1. Viable *Pa* (as a % of all size ranges) in the size range from 0.65 to 3.3 µm captured by ACI stages 4-6 (* no-UV-C test denoted as 0 dose).

UV-C dose (µW s/cm^2)	Median (IQR) Extraction A	Median (IQR) Extraction B	Median (IQR) Extraction C
0*	93.4 (92.7-95.0)	93.3 (91.6-95.2)	93.5 (90.4-96.0)
62	93.1 (90.7-94.7)	93.3 (90.4-93.8)	92.5 (92.2-93.3)
123	94.5 (90.8-96.2)	92.6 (91.2-94.3)	93.0 (92.3-95.3)
246	90.2 (87.6-93.7)	90.2 (85.8-92.7)	93.7 (91.2-96.5)
492	92.8 (89.7-93.7)	92.0 (88.9-93.8)	92.0 (89.8-95.8)
984	89.5 (89.0-92.2)	83.6 (75.1-100.0)	100.0 (60.3-100.0)
1968	93.9 (92.2-94.3)	94.3	100.0 (100.0-100.0)
4920	93.1 (89.8-94.3)	0.0[§]	0.0[§]

[§]CFU=0

Table S2. Mean total decay (%) of airborne *Pa* AUST-02 at 60% RH and their equivalent air exchange rates (eAER) per hour. Note: *no UV-C test denoted as 0 dose, [§]mean natural decay when UV-C was off, [†]for 0 dose tests this is the non-UVC AER due to ventilation and natural decay only.

UV-C dose (µW s/cm^2)	Total n=34	Total decay (%, SD)	Total equivalent air exchange rate (eAER, SD)
0*	8	55.2 (4.4)[§]	2.2 (0.2)[†]
62	3	57.0 (2.4)	2.3 (0.2)
123	4	64.8 (2.6)	2.8 (0.2)
246	4	80.9 (1.4)	4.4 (0.2)
492	4	91.1 (2.9)	6.6 (1.0)
984	4	98.7 (0.9)	12.1 (2.0)
1968	4	99.9 (0.04)	17.8 (0.9)
4920	3	99.9 (0.04)	17.9 (0.9)

Table S3. ANOVA for the difference in UV-C effectiveness (%) between different doses of UV-C against airborne *Pa* AUST-02 at medium RH.

UV-C dose (µW s/cm^2)		Effectiveness	
		Mean difference	p-value
62	123	-20.1	<0.001
	246	-46.3	<0.001
	492	-64.1	<0.001
	984	-77.9	<0.001
	1968	-84.0	<0.001
	4920	-83.0	<0.001
123	246	-26.3	<0.001
	492	-44.1	<0.001
	984	-57.9	<0.001
	1968	-63.9	<0.001
	4920	-62.9	<0.001
246	492	-17.8	<0.001
	984	-31.6	<0.001
	1968	-37.6	<0.001
	4920	-36.6	<0.001
492	984	-13.8	0.001
	1968	-19.9	<0.001
	4920	-18.9	<0.001
984	1968	-6.0	0.365
	4920	-5.1	0.642
1968	4920	1.0	1.000

Table S4. Paired-samples t-test for the difference in the mean total decay (%) (I) and natural decay (J) of airborne *Pa* AUST-02 at medium RH.

UV-C dose (µW s/cm^2)	Total n=26	Mean difference (I-J) (SD)	p-value
62	3	1.8 (2.3)	0.314
123	4	10.4 (2.9)	0.005
246	4	25.4 (3.6)	0.001
492	4	37.5 (2.3)	<0.001
984	4	44.3 (3.8)	<0.001
1968	4	46.6 (4.5)	<0.001
4920	3	43.4 (4.9)	0.004

Table S5. Split plot ANOVA for the effects of RH and UV-C dose on UV-C effectiveness (%).

At 123 µW s/cm^2, UV-C effectiveness increased significantly (p=0.005) at 40% RH compared with 80% RH, while effectiveness was lower at 60% RH than 80% RH, but did not reach significance (p=0.059). At 492 µW s/cm^2, effectiveness at 40% and 60% RH was not significantly different to that at 80% RH. At 1968 µW s/cm^2, effectiveness at 60% was not different to that at 80%, while at 40% RH effectiveness was significantly lower than at 80% RH (p=0.012).

Parameter	Mean (SE)	Mean difference (SE)	p-value
[UV-C dose=123]		-48.8 (8.4)	<0.001
[UV-C dose=492]		-19.2 (7.7)	0.023
[UV-C dose=1968]†		0	
[RH=40] * [UV-C dose=123]	64.4 (4.9)	25.1 (7.7)	0.005
[RH=40] * [UV-C dose=492]	59.6 (6.0)	-9.3 (7.7)	0.242
[RH=40] * [UV-C dose=1968]	64.6 (6.0)	-23.6 (8.4)	0.012
[RH=60] * [UV-C dose=123]	24.6 (4.2)	-14.8 (7.3)	0.059
[RH=60] * [UV-C dose=492]	68.6 (4.2)	-0.3 (6.4)	0.958
[RH=60] * [UV-C dose=1968]	88.5 (4.2)	0.3 (7.3)	0.966
[RH=80] * [UV-C dose=123]‡	39.3 (6.0)	0	
[RH=80] * [UV-C dose=492]§	69.0 (4.9)	0	
[RH=80] * [UV-C dose=1968]#	88.2 (6.0)	0	

Abbreviation: SE, standard error

† UV-C dose reference

‡RH reference at UV-C dose 123

§RH reference at UV-C dose 492

#RH reference at UV-C dose 1968

Table S6. UV-C effectiveness (%) against different genotypes of *Pa* at medium RH.

UV-C dose (µW s/cm^2)	Genotypes	Total n=20	Mean (SD)
123†	ST155	1	35.9
	AUST-01	1	81.1
	AUST-02	4	24.6 (6.1)
492	ST155	1	79.7
	AUST-01	1	86.8
	AUST-02	4	68.6 (2.1)
	AUST-06	1	-3.6*
1968	ST155	1	85.7
	AUST-01	1	87.4
	AUST-02	4	88.5 (2.0)
	AUST-06	1	72.1

Abbreviation: ST155, *Pa* non-clonal strain

†AUST-06 data not recorded (experiment failed)

*AUST-06 results: at extraction B: number of CFU survival of UV-C test was similar to that of no-UV-C test; at extraction C: number of CFU survival of UV-C test was higher than that of no-UV-C test.

Table S7. UV-C effectiveness (%) against airborne *Pa* AUST-02 at different UV-C exposure times at medium RH.

UV-C dose (µW s/cm^2)	Exposure time (min)	Total n=18	Mean (SD)
123	2	1	77.8
	20	4	24.6 (6.1)
	40	1	41.3
492	2	1	92.7
	20	4	68.6 (2.1)
	40	1	73.2
1968	8‡	1	92.1
	20	4	88.5 (2.0)
	40	1	90.8

‡Minimum exposure time at maximum UV-C power level allowed to achieve 1968 µW s/cm^2

Table S8. Basic information of the rooms at TPCH and the assumptions for upper-room UVGI air disinfection estimation.

Type of room	Size (m^3)	ACH Mean ± SD	Height (m)	Lower /upper zone (m)	Distance air travels (m)	No of times air travels in 1 h	Total distance air moves in 1 h	Air velocity (m/min)	Exposure time at the upper 0.675 m UV-C zone (min)
Lung function room	168.5	8.5 ± 0.8	2.7	1.35	2.025	17	34.4	0.57	1.18
Consultation room A	31.5	7.0 ± 0.1	2.7	1.35	2.025	14	28.4	0.47	1.43
Consultation room B	36.0	6.1 ± 0.1	2.7	1.35	2.025	12.2	24.7	0.41	1.64

Table S9. The upper room exposure time and the minimum average UV-C intensity required that correspond to the experimental results of total reduction of airborne *Pa* AUST-02.

UV-C dose (μW s/cm^2)	UV-C effectivenes s (%)	Lung function room		Consultation room A		Consultation room B	
		Exposure time (min)	Required UV-C intensity (μW/cm^2)	Exposure time (min)	Required UV-C intensity (μW/cm^2)	Exposure time (min)	Required UV-C intensity (μW/cm^2)
62	4.5	1.18	0.87	1.43	0.72	1.64	0.63
123	24.6	1.18	1.74	1.43	1.43	1.64	1.25
246	50.9	1.18	3.47	1.43	2.87	1.64	2.50
492	68.6	1.18	6.95	1.43	5.73	1.64	5.00
984	82.5	1.18	13.90	1.43	11.47	1.64	10.00
1968	88.5	1.18	27.80	1.43	22.94	1.64	20.00
4920	87.5	1.18	69.49	1.43	57.34	1.64	50.00

Table S10. The eye level estimated UV-C intensity required that correspond to the experimental results of total reduction of airborne *Pa* AUST-02.

UV-C dose (μW s/cm^2)	UV-C effectiveness (%)	Lung function room		Consultation room A		Consultation room B	
		Eye-level UV-C intensity (μW/cm^2)	% Occupational exposure limit	Eye-level UV-C intensity (μW/cm^2)	% Occupational exposure limit	Eye-level UV-C intensity (μW/cm^2)	% Occupational exposure limit
62	4.5	0.004	3	0.003	3	0.003	2
123	24.6	0.008	6	0.007	5	0.006	5
246	50.9	0.016	12	0.013	10	0.012	9
492	68.6	0.031	24	0.027	21	0.024	18
984	82.5	0.063	49	0.054	42	0.047	37
1968	88.5	0.126	98	0.108	84	0.094	73
4920	87.5	0.314	245	0.269	210	0.236	184

Figure S1. Outline of the experiments.

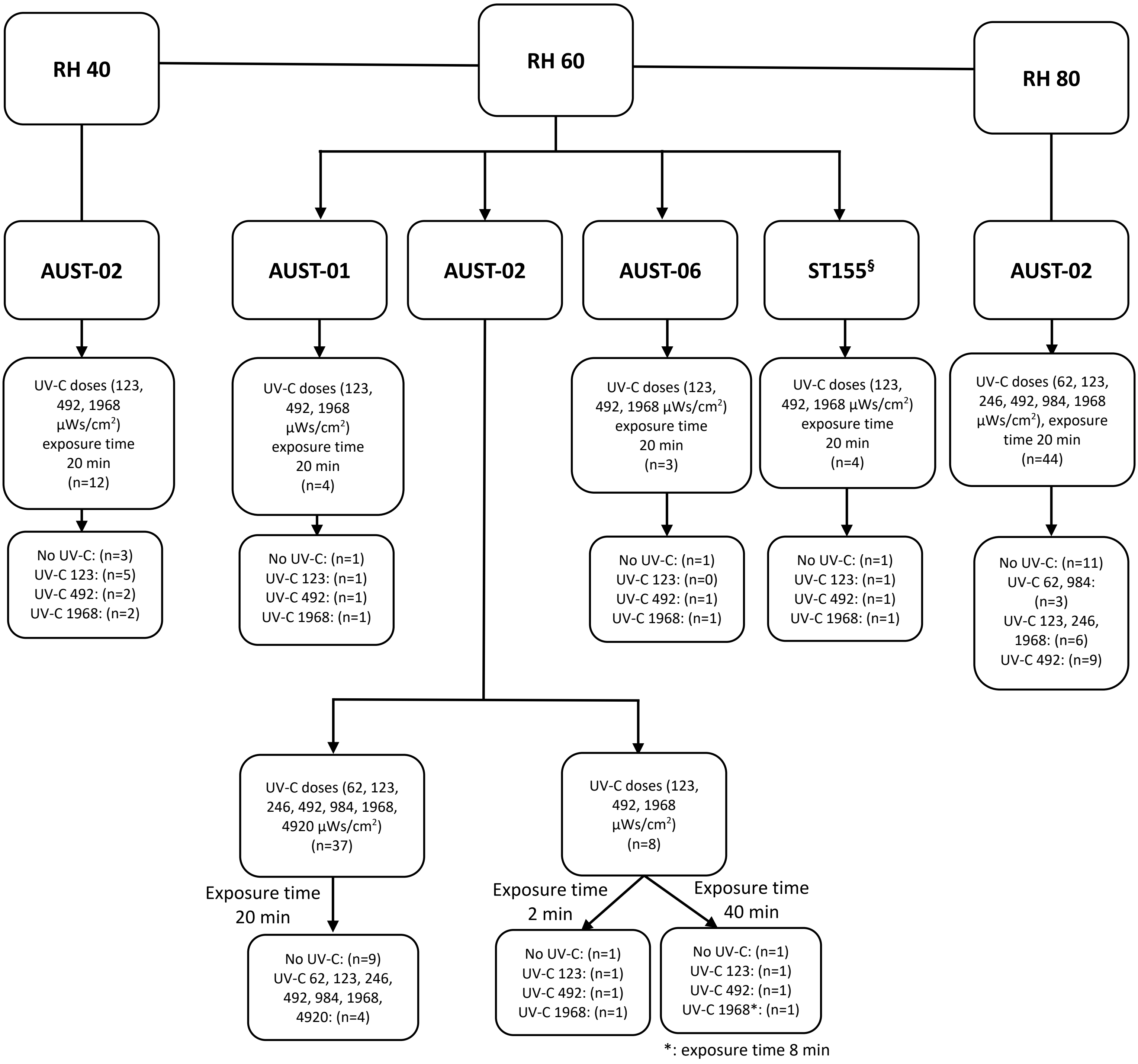

§*Pa* non-clonal strain

Figure S2. Box plots of the natural decay (%) of aerosolized *Pa* AUST-02 by extraction time and RH. Note: extractions B and C commenced at 30 min and 40 min, respectively, after the initial nebulization and ran for 5 min each. Compared with high RH, decay was more rapid at low RH (p=0.001) and medium RH (p=0.015).

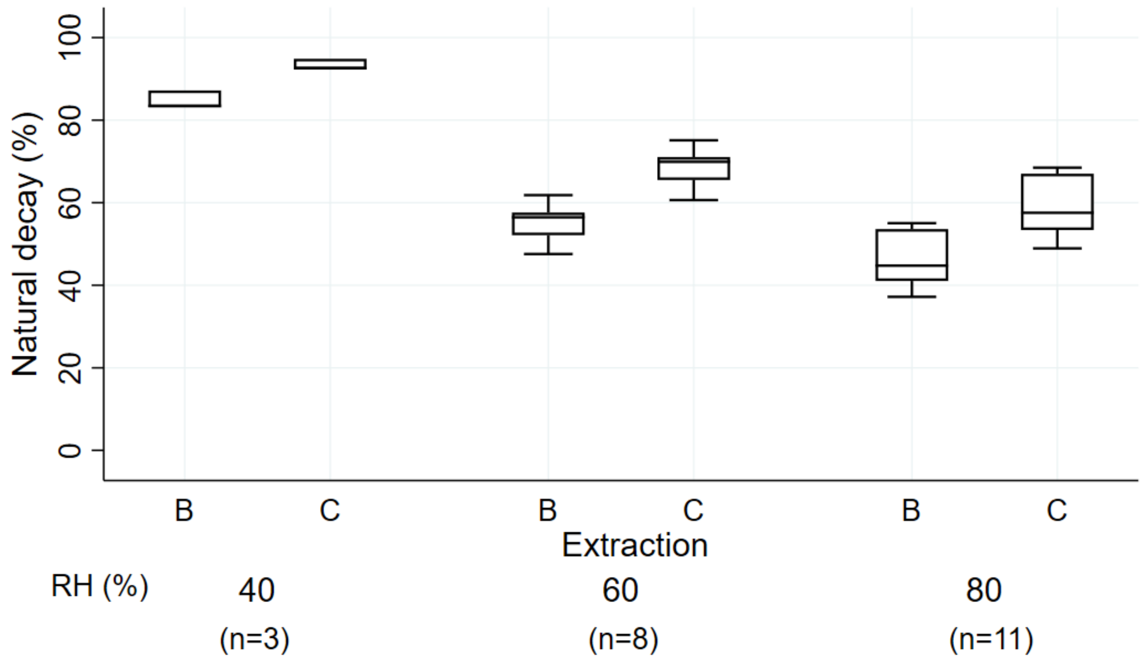

Figure S3. Particle concentrations and size distribution inside the drum at medium RH.

Extraction A

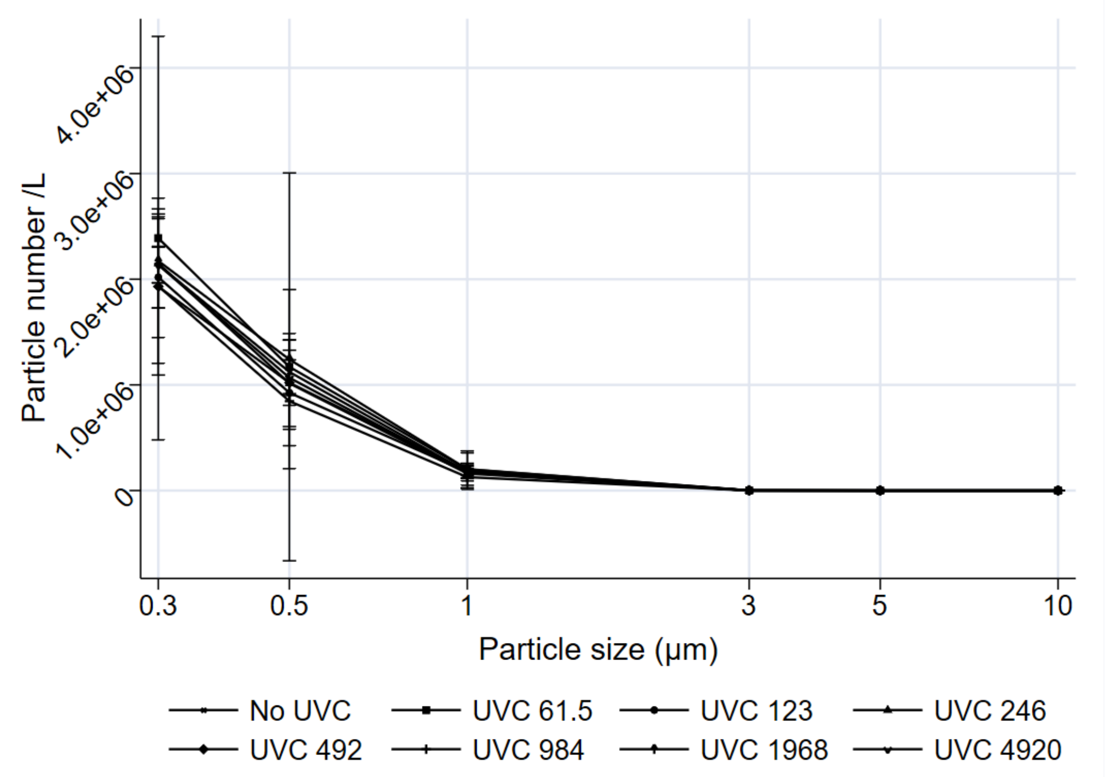

Extraction B

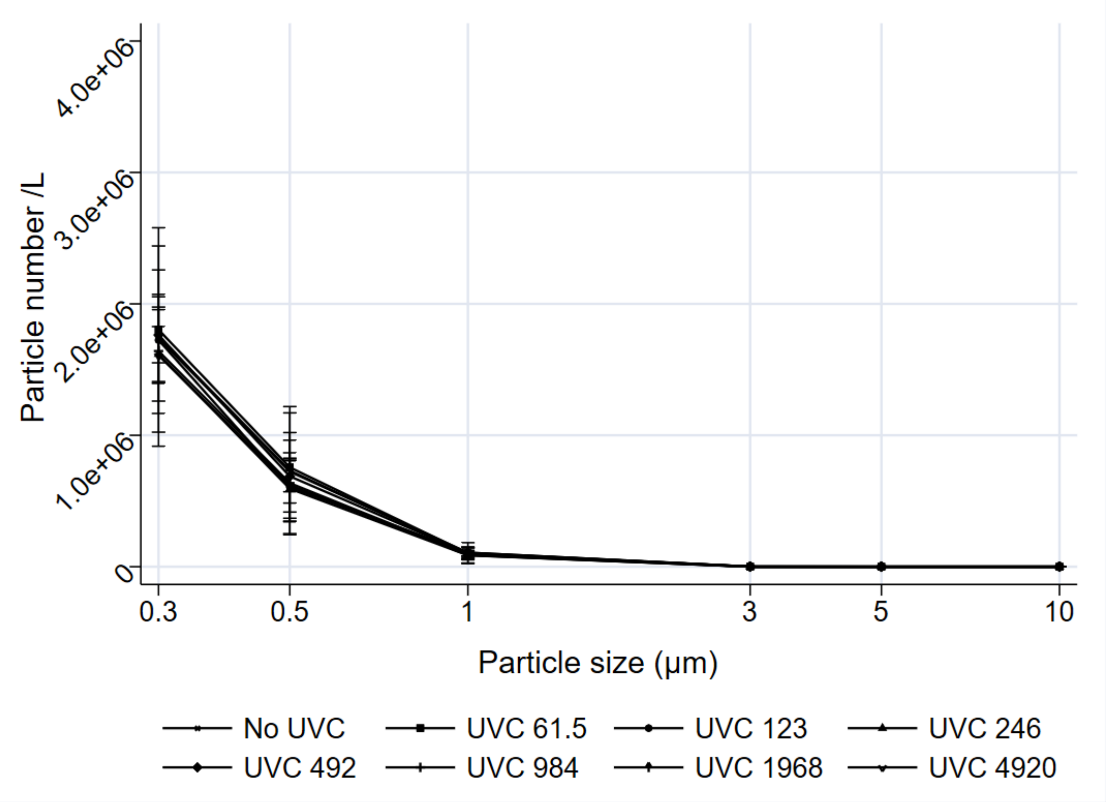

Extraction C

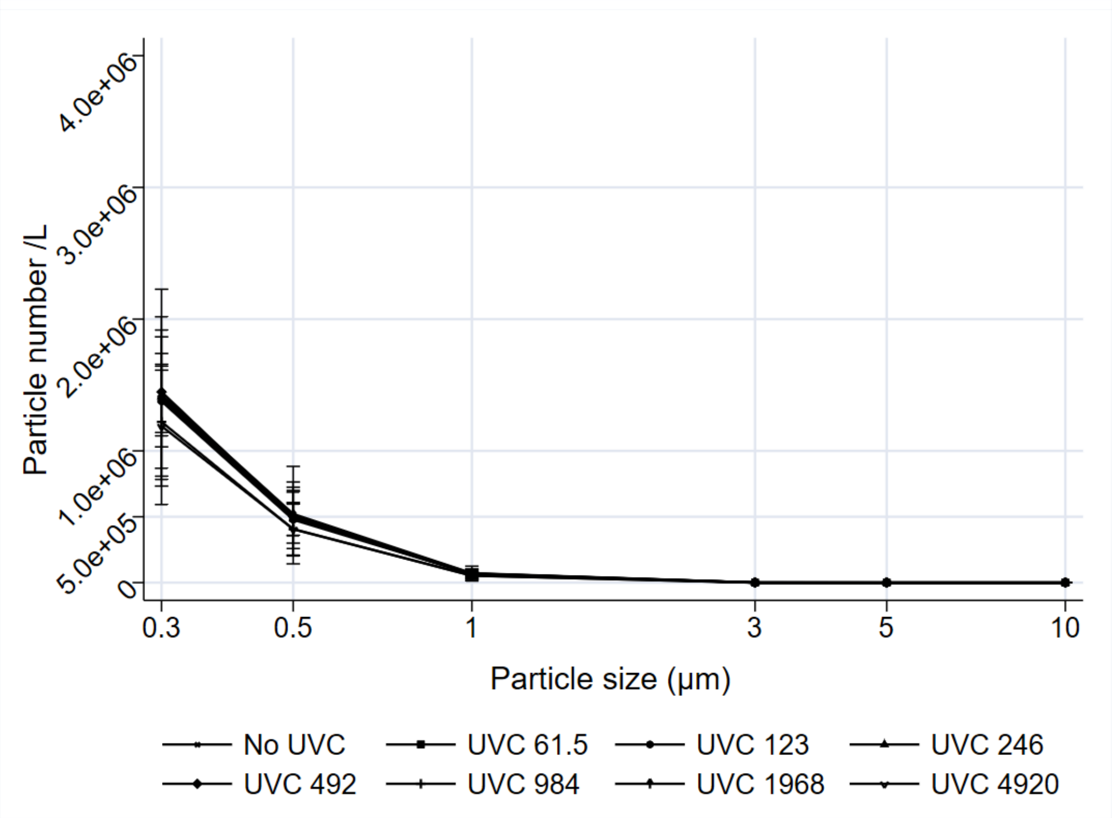

Reference

1. Kidd TJ, Ramsay KA, Hu H, Marks GB, Wainwright CE, Bye PT, et al. Shared Pseudomonas aeruginosa genotypes are common in Australian cystic fibrosis centres. Eur Respir J. 2013;41(5):1091-100. Epub 2012/08/11. doi: 10.1183/09031936.00060512. PubMed PMID: 22878877.

2. Knibbs LD, Johnson GR, Kidd TJ, Cheney J, Grimwood K, Kattenbelt JA, et al. Viability of Pseudomonas aeruginosa in cough aerosols generated by persons with cystic fibrosis. Thorax. 2014;69(8):740-5. Epub 2014/04/20. doi: 10.1136/thoraxjnl-2014-205213. PubMed PMID: 24743559; PubMed Central PMCID: PMCPMC4112489.

3. Knibbs LD, Morawska L, Bell SC, Grzybowski P. Room ventilation and the risk of airborne infection transmission in 3 health care settings within a large teaching hospital. American Journal of Infection Control. 2011;39(10):866-72. doi: 10.1016/j.ajic.2011.02.014. PubMed PMID: WOS:000298137100011.

4. Brickner PW, Vincent RL, First M, Nardell E, Murray M, Kaufman W. The application of ultraviolet germicidal irradiation to control transmission of airborne disease: bioterrorism countermeasure. Public Health Rep. 2003;118(2):99-114. Epub 2003/04/12. doi: 10.1093/phr/118.2.99. PubMed PMID: 12690064; PubMed Central PMCID: PMCPMC1497517.
